# A promise for neuronal repair: reprogramming astrocytes into neurons *in vivo*

**DOI:** 10.1042/BSR20231717

**Published:** 2024-01-25

**Authors:** Lijuan Huang, Xinyu Lai, Xiaojun Liang, Jiafeng Chen, Yue Yang, Wei Xu, Qingchun Qin, Rongxing Qin, Xiaoying Huang, Minshan Xie, Li Chen

**Affiliations:** 1 Department of Neurology, the First Affiliated Hospital, Guangxi Medical University, Nanning, 530021, China; 2 State Key Laboratory of Targeting Oncology, National Center for International Research of Bio-Targeting Theranostics, Guangxi Key Laboratory of Bio-Targeting Theranostics, Collaborative Innovation Center for Targeting Tumor Diagnosis and Therapy, Guangxi Medical University, Nanning, Guangxi, 530021, China; 3 Collaborative Innovation Centre of Regenerative Medicine and Medical BioResource Development and Application Co-constructed by the Province and Ministry, Guangxi Medical University, Nanning, Guangxi, 530021, China

**Keywords:** astrocytes, neurodegeneration, neuroregeneration, transcription factors

## Abstract

Massive loss of neurons following brain injury or disease is the primary cause of central nervous system dysfunction. Recently, much research has been conducted on how to compensate for neuronal loss in damaged parts of the nervous system and thus restore functional connectivity among neurons. Direct somatic cell differentiation into neurons using pro-neural transcription factors, small molecules, or microRNAs, individually or in association, is the most promising form of neural cell replacement therapy available. This method provides a potential remedy for cell loss in a variety of neurodegenerative illnesses, and the development of reprogramming technology has made this method feasible. This article provides a comprehensive review of reprogramming, including the selection and methods of reprogramming starting cell populations as well as the signaling methods involved in this process. Additionally, we thoroughly examine how reprogramming astrocytes into neurons can be applied to treat stroke and other neurodegenerative diseases. Finally, we discuss the challenges of neuronal reprogramming and offer insights about the field.

## Introduction

In the central nervous system (CNS) of adult mammals, a number of neural stem cells are present in the subventricular and subgranular regions of the hippocampal dentate gyrus [1]. After brain injury, these neural stem cells give rise to neuroblasts and migrate to the site of injury thereby generating new neurons [2,3]. However, neuronal repair and regeneration in the context of brain injury and neurological disorders remains a challenge. Over the last decade, researchers have aimed to develop cell replacement therapies that can effectively replace lost neurons. A promising approach is cellular reprogramming, which involves the use of various methods, such as delivering small molecules or ectopically expressing transcription factors and microRNAs to manipulate cell fate. This nascent field has demonstrated considerable potential in several studies. In 2006, Takahashi and Yamanaka demonstrated that somatic cells could be reprogrammed into induced pluripotent stem cells (iPSC) by manipulating four transcription factors: *OCT4*, *SOX2*, *KLF4*, and *c-MYC*. This approach led to the development of iPSC transplantation therapy, which is considered an effective strategy in regenerative medicine [4]. However, there are several challenges related to this methodology, such as ethical concerns, tumorigenicity, and abnormal differentiation that limit the clinical application of iPSCs. An intriguing alternate approach to address these issues is the direct conversion of CNS cells into new neurons without passing through intermediate states of pluripotency or multipotency. This process is known as ‘transdifferentiation’. This *in situ* neuronal generation by direct reprogramming holds great promise in regenerative medicine [5]. In addition to neurons, the most abundant cells in the CNS are glial cells, including astrocytes and oligodendrocyte precursor cells (OPC); these cells are also known as NG2 glia and microglia [6,7]. Previous studies have shown that astrocytes, OPC, and microglia can all be reprogrammed into neurons *in vivo* [8–11]. Here, we mainly discuss astrocyte mediated reprogramming. Astroglial cells are the most common cell type in the adult mammalian CNS (approximately 30% of all cells) [12] and share primitive progenitor cells with neurons; they are prime candidates for transformation into neurons [13]. Compared with iPSC reprogramming, the direct lineage conversion of astrocytes into induced neurons (iNs) offers an alternative means of generating functional neurons *in vivo* while avoiding the complications associated with CNS transplantation, making it a promising therapy for brain repair. This review outlines the selection of cells and initiation methods for reprogramming, describes the signaling pathways involved in the process, and discusses the potential applications of reprogramming in neurological diseases such as stroke. Finally, challenges in the field of reprogramming are addressed, and future research prospects are proposed.

## Astrocytes are optimal cells for reprogramming

The choice of the starting cell type is an important step in direct reprogramming, based on the principle that the number of starting cells is sufficient and that the starting cells are easy to reprogram. In the field of reprogramming, fibroblasts are one of the most often employed starting cells due to their quantity and accessibility of materials, including fibroblasts from mouse or human skin. The first direct transdifferentiation using mouse fibroblasts was achieved by overexpressing three transcription factors, *Ascl1*, *Brn2*, and *Myt1l* (BAM factors) [14]. However, reprogramming fibroblasts into neurons requires transdifferentiation across lineages, which increases the difficulty of reprogramming. Additionally, fibroblast-transformed neurons must be transplanted into the brain, facing the obstacle of immune rejection. Therefore, scientists have targeted the widespread distribution of astrocytes in the CNS, which share a common origin with neurons originating from radial glial cells [13]. Additionally, astrocytes play an important role in maintaining brain stability, can add value after CNS damage, activate reactive astrocytes, and acquire neurogenic potential [15]. Reactive astrogliosis is a protective response against toxic substances that can damage the brain. However, it can also lead to neuroglial scarring, which impedes brain tissue regeneration, further contributing to neurodegenerative processes. Hence, controlling reactive astrogliosis is crucial to preventing further brain damage and promoting brain repair [16]. Thus, reprogramming astrocytes into neurons not only obviates the risk of immune rejection caused by allografts but also specifically inhibits the formation of glial scars and promotes neurological recovery by replenishing neurons lost due to injury. Therefore, astrocyte transdifferentiation has received increasing attention as a therapeutic strategy for neuronal replacement following brain injury.

## Reprogramming methods

### Transcription factors

**Figure 1:**
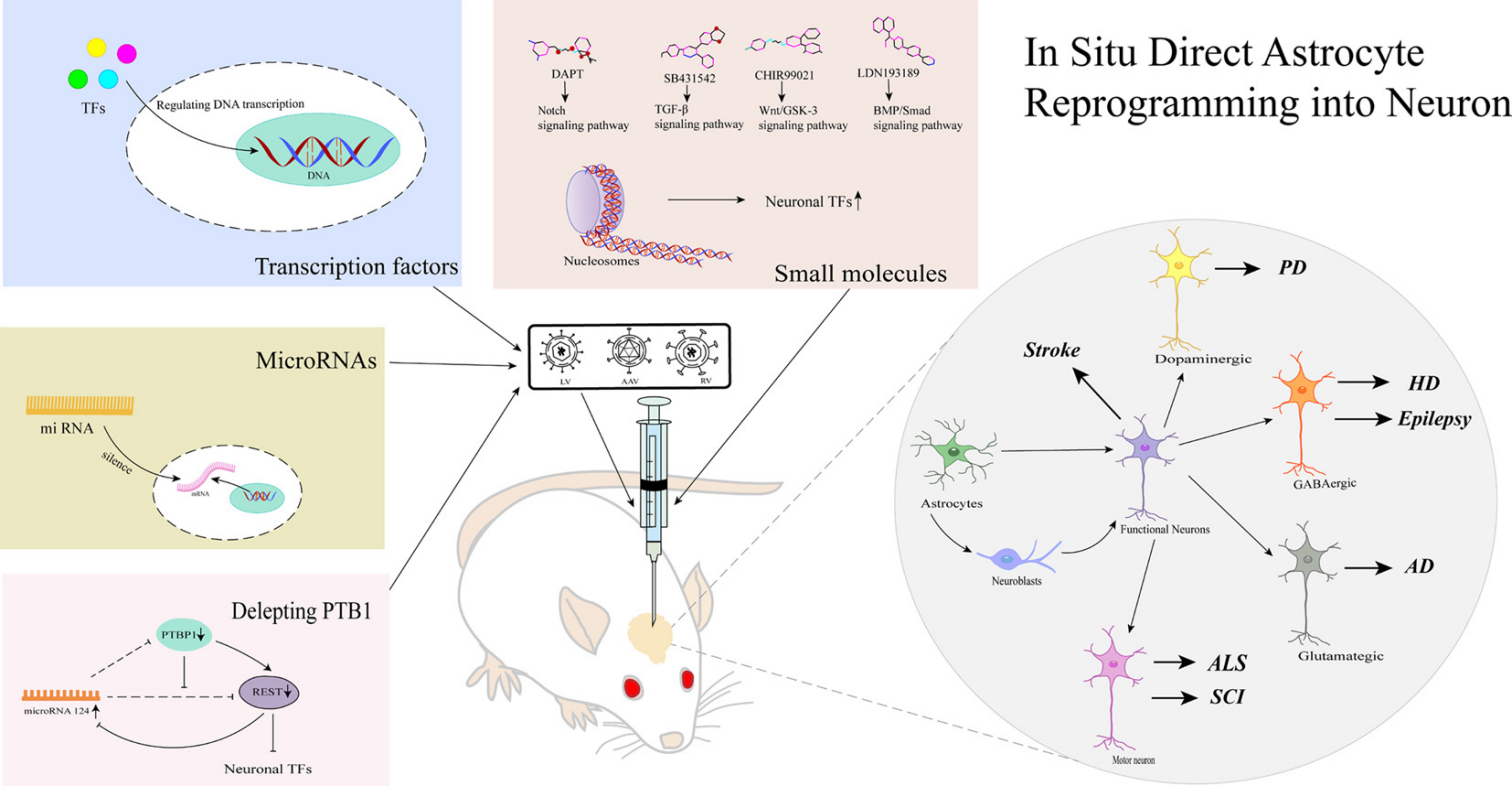
Methods for reprogramming astrocytes into neurons *in vivo* *In vivo* astrocyte reprogramming methods include the use of micro syringes, injection of AAV, LV, or RV carrying different transcription factors and microRNAs, direct injection of small molecule compounds into the cerebral cortex, striatum, or other CNS tissues, or direct deletion of PTBP1. Transcription factors regulate the transcriptional activity of genes by binding to the DNA. A small molecule cocktail regulates chromatin by targeting different signaling pathways, ultimately leading to an increase in neural transcription factor activity. microRNAs are regulated by silencing mRNA produced by transcription. Deletion of the PTBP1 gene represses the expression of complex REST and miR-124 by targeting silencing of transcription factors, leading to the expression of neuron-specific transcription factors in neurogenesis. There are two types of reprogramming: direct reprogramming, in which cells directly transdifferentiate into neurons without passing through the adult neuronal cell or immature state; and indirect reprogramming, in which cells pass through the adult neuronal cell or immature neuronal cell state and then further differentiate into neurons in response to other stimuli. *In situ* injection of viral vectors or small molecule mixtures containing target genes to reprogram astrocytes into different subtypes of neurons *in vivo* is used in the treatment of some CNS diseases. Abbreviations: AAV, adeno-associated viruses; AD, Alzheimer’s disease; ALS, amyotrophic lateral sclerosis; HD, Huntington’s disease; LV, lentiviruses; PD, Parkinson’s disease; RV, retroviruses; SCI, spinal cord injury; TF, transcription factors.

Reprogramming techniques have advanced to the point where astrocytes can be converted into neurons, both *in vitro* and *in vivo*, by overexpressing one or more crucial transcription factors (Table 1). These developments have the potential to promote brain repair and overcome neurodegeneration. In 2002, by overexpressing a single transcription factor, Paired box 6 (Pax6), Heins et al. were first to direct glial cells toward neurons using cultured astrocytes [17], opening the door for the reprogramming of astrocytes into neurons. During embryonic development, Neurogenin2 (*Ngn2*) drives the development of excitatory glutamatergic neurons in the dorsal telencephalon, and efficient conversion of astrocytes into glutamatergic neurons by retroviral overexpression of *Ngn2* [18,19]. In contrast, few reprogrammed neurons are produced *in vivo* by *Ngn2* overexpression alone, and several research groups have shown that *Ngn2* needs to be combined with other factors, such as the anti-apoptotic protein Bcl-2 or the antioxidant vitamin E [20], and the growth factors EGF and FGF [21] to efficiently reprogram cells *in vivo* (Figure 1). This suggests that even factors that are able to reprogram cells efficiently *in vitro* do not necessarily exhibit the same efficiency *in vivo*, highlighting the differences in these distinct reprogramming conditions. Furthermore, combining multiple transcription factors has been previously demonstrated to guide astrocytes to differentiate into specific neuronal subtypes. Recently, Zhou et al. made a breakthrough in this field using the CRISPR-Cas transcriptional activation (CRISPRa) system, which enables the transformation of spinal cord astrocytes into functional motor neurons capable of establishing synaptic connections [22].

**Table 1:** Summary of different transcription factors reprogramming astrocytes into different subtypes of neurons and conversion efficiency

Species	Disease model	Source cell	TFs	Vectors used	Transformed cell type	Transduction efficiency	Reference
Mouse	Cerebral ischemic injury	Cortex astrocytes	NeuroD1	AAV	NeuN +	33%	[17]
Monkey	Cerebral ischemic injury	Cortex astrocytes	NeuroD1	AAV	NeuN +	94.4 ± 5.5%	[18]
Mouse	Cerebral ischemic injury	Cortex astrocytes	Ngn2+Bcl-2	LV	NeuN +	Very low, <1%	[19]
Mouse	Cerebral ischemic injury	Cortex astrocytes	NeuroD1	AAV	NeuN neuron with BDNF/NOG/VPA	Unknown	[20]
Mouse	PD	Striatal astrocytes	NeuroD1, Ascl1, Lmx1a, miR218e	LV	DCX+ or RBFOX3+	Unknown	[21]
Mouse	HD	Striatal astrocytes	NeuroD1 and Dlx2	AAV	GABAergic neurons	78.60%	[22]
Mouse	AD	Cortex astrocytes	NeuroD1	RV	Glutamatergic neurons	92.8 ± 2%	[23]
Rat	TLE	Hippocampal astrocytes	NeuroD1	AAV	GABAergic neurons	88.5%	[24]
Mouse	Epilepsy	Hippocampal astrocytes	Ascl1 and Dlx2	RV	GABAergic neurons	70%	[25]
Mouse	SCI	Spinal cord astrocytes	Sox2	LV	DCX+	6–8%	[26]
Mouse	SCI	Spinal cord astrocytes	Sox2	AAV	TUJl+	22.10%	[27]
Mouse	SCI	Spinal cord astrocytes	NeuroD1	LV	NeuN+, DCX+, and Nestin+	Unknown	[28]
Mouse	SCI	Spinal cord astrocytes	NeuroD1	AAV	Glutamatergic neurons	95%	[29]

Abbreviations: AAV, retrovirus; Ascl1, achaete-scute family bHLH transcription factor 1; Bcl-2, B-cell lymphoma-2; DCX, doublecortin; Dlx2, distal-less-2; LV, lentiviral; NeuroD1, neuronal differentiation 1; Ngn2, neurogenin2; RV, retrovirus; Sox2, SRY-box transcription factor 2; TUJ1, class III β-tubulin.

*Ascl1* and *Dlx* are thought to be determinants of the fate of inhibitory GABAergic neurons in the ventral telencephalon [28], and it has been shown that *Ascl1* converts astrocytes in the cortex and striatum into neurons not only *in vitro*, but also *in vivo*, and that these iNs can generate functional synapses. Interestingly, astrocytes in different brain regions are converted with different efficiencies; however, the mechanism underlying this heterogeneity in regional conversion efficiency remains unclear [29]. *Dlx2* alone induces reprogramming only approximately 30% of the time to generate new neurons but combining it with *Ascl1* increases reprogramming efficiency [19,30]. The process of converting striatal astrocytes to GABAergic cells by *Dlx2* is similar to how neural stem cells develop, according to single-cell RNA sequencing (scRNA-seq) [31].

Through retroviral targeting of proliferating reactive glial cells after brain stab injury, Guo et al. demonstrated that *NeuroD1*, which encodes a basic helix-loop-helix (bHLH) transcription factor, effectively reprogrammed reactive astrocytes into glutamatergic iNs. Additionally, membrane-clamp recordings have shown that these iNs are capable of repeatedly generating action potentials and receiving synaptic inputs [32]. Moreover, *NeuroD1* induced astrocyte-to-neuron reprogramming has been used in different disease models, such as stroke [23], Parkinson’s disease (PD) [33], Huntington’s disease (HD) [34], Alzheimer’s disease (AD) [32], epilepsy [35], and spinal cord injury (SCI) [36] (Table 1), triggering different translational efficiencies and subtypes of reprogrammed neurons that not only generate functional synapses, but also rescue behavioral deficits and cognitive dysfunction in some animals.

Zhang et al. demonstrated an alternative approach to reprogramming astrocytes by inducing the generation of proliferating DCX-positive neuroblasts in the striatum via overexpression of the transcription factor *Sox2*. The neuroblasts were then transformed into functional neurons by the addition of brain-derived neurotrophic factor (BDNF) and the bone morphogenic protein (BMP) inhibitor Noggin (NOG) (Figure 1) [37]. They also discovered that *Sox2* was used to reprogram adult mouse spinal cord astrocytes into neuroblasts, which subsequently differentiated into mature neurons after treatment with Valproic acid (VPA) [38]. Conversion of astrocytes into neuroblasts is a promising strategy for generating a large pool of neuroblasts within the brain. However, neuroblast-to-neuron differentiation requires additional assistance and is inefficient in the adult brain. To improve conversion efficiency, it is important to investigate new combinations of small molecules. Further studies are needed to optimize this process and enhance its potential for application in regenerative medicine.

Transcription factor-mediated reprogramming is currently the most popular and efficient reprogramming method. However, it has several limitations, including the potential for insertional mutations, transgene reactivation, and prolonged gene expression. Therefore, alternative, safe, and effective cell reprogramming methods must be developed to obtain functional neurons for clinical therapy.

### Small chemical molecules

Reprogramming astrocytes into neurons using small-molecule drugs is a promising alternative to transcription factor-based methods because of their low cost, ease of production, and the absence of genomic integration. In addition, unintended changes in intracellular targets caused by small molecules make them effective cell fate determinants *in vivo*. This could facilitate tissue repair following CNS injury by enabling the regenerated cells to assume the desired phenotype (Figure 1). In 2015, Pei et al. used the small-molecule compound ‘VCR’ cocktail (VPA, CHIR99021, RepSox) to directly convert mouse astrocytes into functional neurons [39] (Table 2). The conversion efficiency was improved by the addition of ISX-9, i-Bet151, and Forskolin. The transformed neurons remained viable when transplanted into mice [40] (Table 2). Furthermore, other researchers converted fetal astrocytes into glutamatergic neurons by sequentially adding LDN193189, SB431542, TTNPB, Tzv, CHIR99021, VPA, DAPT, SAG, and Purmo. After implantation into the mouse brain, these iNs not only survived but were also able to integrate into the mouse neural network [41]. To simplify the combination of small molecules, Chen et al. transdifferentiated human fetal astrocytes into functional neurons using only DAPT, SB431542, LDN193189, and CHIR99021 or their functional analogs [42] (Table 2). In addition, Ma et al. found that a chemically defined ‘FICB’ cocktail, including Forskolin, ISX-9, CHIR99021, and I-BET151, induced *in vitro* conversion of mouse astrocytes to neurons. In addition to increasing the effectiveness of neuron conversion, the ‘DFICBY’ combination formed by the addition of DBcAMP and Y-27632 also efficiently transformed mouse striatal astrocytes into cortical glutamatergic neurons and GABAergic neurons [43] (Table 2). The chemical conversion of neurons *in vivo* was also achieved.

**Table 2:** Summary of small molecule cocktails directly reprogramming astrocytes into neurons and conversion efficiency

Source cells	Small molecules	iNs	Neuronal induction duration	Efficiency of reprogramming	References
Human astrocytes	LDN193189, SB431542, TTNPB, Tzv, CHIR99021, VPA, DAPT, SAG, Purmo	TUJ1+, DCX+, MAP2+, NeuN+, SYN1+, GABA+, vGLUT1+	8 days	68.7% ± 4.2% NeuN+ at day 8, 88.3% ± 4% vGLUT1+ at 2M, 8.2% ± 1.5% GAD67+ at 2M,	[42]
Mouse astrocytes	VPA, CHIR99021, Repsox	DCX+, TUJ1+, NeuN+	12 days	32% DCX+ at day 12, 24% NeuN+ at day 18	[43]
Human astrocytes	VPA, CHIR99021, Repsox, Forskolin, i-BET151, ISX-9	DCX+, MAP2+, TUJ1+, NeuN+, GABA+, vGLUT1+	24 days	70% vGLUT1+ at day 40, 4% CHAT+ at day 40	[44]
Human fetal astrocytes	DAPT, CHIR99021, SB431542, LDN193189	DCX+, TUJ1+, MAP2+, GAD67+, TH+	6 days	78% VGlut1+ at 3M, 2% GAD67+ at 3M, 1% TH+ at 3M	[45]
Human astrocytes/Mouse astrocytes	Kenpaullone, Forskolin, Y-27632, purmorphamine, retinoic acid,	TUJ1+, MAP2+, NeuN+, SYN1+, HB9+, ISL1+, CHAT+, VAChT+	7 days	93.7 ± 1.6% TUJ1+ at day 14	[46]
Mouse astrocytes	Forskolin, ISX-9, CHIR99021, i-BET151, DBcAMP, Y-27632	Tau+, TUJ1+, MAP2+, SYN1+, NeuN+, GABA+, vGLUT1+	16 days	91.1% ± 2.6% Tau+ at day 16, 94.1% ± 4.1% NeuN+ at day 14, 88.2% ± 2.7% TUJ1+ at day 16	[47]
Mouse astrocyte C8-D1a cell line	SAG, CHIR99021, DAPT, Ruxolitinib, RepSox, Y-26732	TUJ1+, DCX+, MAP2+, NeuN+, vGLUT1+, GAD67+, TH+	4 days	82 ± 6% TUJ1+ at day 4	[48]

M, month.

These results indicate that the reprogramming of astrocytes into neurons can be successfully achieved using small chemical molecules, opening new avenues for reprogramming astrocytes into neurons. However, the development of small molecule combinations that can successfully reprogram astrocytes may necessitate a thorough comprehension of each compound’s mechanism of action and kinetics, necessitating a lengthy period of time for additional screening and the determination of optimal concentrations. Additionally, different combinations induce different neuronal properties and transformation efficiencies, which also requires further investigation. Most importantly, owing to the complex microenvironment *in vivo*, the delivery of small molecules to the brain in a non-invasive manner and maintenance of constant concentrations remain the biggest challenges for clinical applications of these methods today.

### microRNAs

Both transcription factors and non-coding RNAs play important roles in the reprogramming of astrocytes into neurons, but they have different mechanisms and targets; the former mainly regulates gene expression at the transcriptional level, whereas the latter mainly regulates gene expression post-transcriptionally, acting mainly on mRNA transcripts encoding proteins [44]. Because of their small size, microRNAs can be delivered more efficiently into cells than transcription factors encoded by DNA or mRNA. In contrast with traditional DNA-based cellular reprogramming approaches, microRNAs are key regulators of reprogramming efficiency, regardless of genomic integration of foreign nucleic acids. Thus, microRNA-mediated cell reprogramming may be an alternative and potentially safe method of producing reprogrammed cells. A microRNA referred to as miR-124 has important effects on neurogenesis and regulation of neuronal activity, ultimately affecting neuronal cell fate and development. One of the important target genes of miR-124 is REST [45], a transcriptional repressor that inhibits the transcription of neuronal genes in non-neuronal cells; its targeted regulation is essential for maintaining neuronal-specific gene expression and normal neuronal function [46]. Researchers discovered that miR-124 accelerated the transformation of rat cortical reactive astrocytes into cholinergic and glutamatergic neurons when combined with the small molecule medications rucotinib, SB203580, and Forskolin [47]. The expression level of miR-124 was increased throughout this process, hastening the transformation of astrocytes into neurons.

Recent studies have shown that miR-302/367 play an important role in neuronal transformation. Researchers successfully transformed adult mouse cortical astrocytes into neuroblast cells using miR-302/367 both *in vitro* and *in vivo*. However, the reprogramming of adult mouse striatal astrocytes into neuroblast cells requires the addition of sodium valproate, which has been widely used in cell reprogramming studies to promote neuronal transformation. Overall, the discovery of miR-302/367 provides new insights and methods for neuronal transformation and regeneration, and the application of auxiliary substances, such as sodium valproate, is expected to further improve cell transformation efficiency [48]. In addition to these microRNAs, it was also shown that miR-365 knockdown effectively increased *Pax6* expression in responsive astrocytes and promoted the conversion of astrocytes into neurons [49]. In PD models, *Ascl1* / *Lmx1a* / *NeuroD1* co-expression with miR-218 converts striatal astrocytes into dopaminergic neurons, which not only restores dopaminergic transmission but also partially restores functional defects in PD mouse models [27]. Overall, miRNAs are receiving increased attention for their potential to refine direct reprogramming of cells into neurons.

However, reprogramming is a complicated process, and microRNAs alone are insufficient for cellular transdifferentiation; transcription factors or small molecules must also be involved. These molecules interact in a complex feedback loop to facilitate the transdifferentiation of astrocytes into neurons [50]. Therefore, examining the relationships between microRNAs, transcription factors, and small molecules is crucial for understanding astrocyte-to-neuron transformations. Furthermore, there is no better way to enhance the half-life of microRNA and facilitate its translation into the body. In short, further studies are needed to fully understand the mechanism of microRNA-mediated cellular reprogramming.

### Depleting PTBP1

Recent studies have shown that in addition to transcription factors, small chemical molecules, and microRNAs, inhibition of the RNA-binding protein pyrimidine binding protein 1 (PTBP1), induces the involvement of PTBP1 in the regulation of gene expression and variable splicing and promotes the conversion of adult astrocytes to neurons (Table 3). Research has demonstrated that PTBP1 controls the splicing of genes in neurons throughout the development of the brain, and that when PTBP1 expression is reduced, specific RNA splicing in developing neurons is encouraged by increased PTBP2 expression. Furthermore, the promotion of certain neuronal genes in non-neuronal cells is facilitated by the down-regulation of PTBP1 and the neuron-specific microRNA miR124, which targets the neuronal transcriptional repressor REST. In a mouse model of 6-hydroxydopamine-induced PD, by injecting AAV9-Gfap-mCherry and CasRx-Ptbp1 constructs into the ipsilateral striatum of PD mice, Qian et al. delivered an RNA-silencing hairpin (shRNA) using AAV2 *in vivo* to reduce PTB1 expression in astrocytes of GFAP-Cre mice. The resulting striatal astrocytes were reprogrammed into neurons and displayed mature synaptic features, thereby rescuing the motor phenotype shown in PD mice [51]. Furthermore, Zhou et al. knocked down PTBP1 and induced the expression of mCherry-positive dopaminergic neurons. This effectively improved the PD mice's symptoms and confirmed the physiological activity of the induced neurons by membrane clamp [52]. Moreover, Maimon et al. used old and normal mice models to promote transdifferentiation of astrocytes to neurons using ASO-mediated PTBP1 knockdown. The resultant neurons exhibited neuronal markers and produced action potentials [53]. However, many doubts have lately been expressed concerning the induced neurons generated by PTPB1 knockouts, indicating that viral leakage rather than astrocytic glial cell transdifferentiation is the cause of these ‘nascent’ neurons [54,55]. Despite this controversy, neuronal repair and regeneration research remain ongoing. Further research is required to clarify the precise mechanisms by which PTBP1 silencing accomplishes the glial-to-neuronal transformation and to solve the issue of neuronal mislocalization by means of meticulous lineage tracing.

**Table 3:** Summary of direct reprogramming of astrocytes into neurons by PTBP1 knockout

Species	Disease model	Starting cell type	Vector/Delivery system	Transformed cell type	Induction efficiency	References
Mouse	PD	Midbrain astrocytes	AAV-shPTBP1	Dopaminergic	35%	[56]
Mouse	PD	Striatum astrocytes	AAV with gRNAs targeting PTBP1	Glutamatergic	∼50%	[57]
Mouse	Normal adult and age mouse	Dentate gyrus GFAP^+^ cells	ASO-PTBP1 CSF injection	NeuN +	15%	[58]

PD, Parkinson’s disease.

## Critical signaling pathways involved in astrocyte reprogramming

The CNS is regulated by multiple external signaling pathways in addition to intrinsic genetic programming during development [57]. Similarly, the reprogramming of astrocytes into neurons is regulated by multiple signaling pathways, and identifying these pathways would enable researchers to directly control important signaling inside the cell through small molecules. Consequently, noninvasive neural change would be possible. In a recent study, researchers used small molecule compounds to regulate several major signaling pathways, including Notch, glycogen synthase kinase 3b (GSK-3b), transforming growth factorβ (TGF-β), and BMP to induce the conversion of astrocytes into functional neurons [42]. It was found that REST expression was significantly decreased during small molecule reprogramming, leading to overexpression of *NeuroD1, Ngn2*, and other transcription factors, which activated the reprogramming process [58].

### Notch signaling pathway

The Notch signaling pathway is a highly conserved signaling pathway that plays a key role in regulating multiple neuronal differentiation decisions during neurodevelopment and adult neurogenesis [59]. Notch signaling receptors in mammals induce the release of the Notch intracellular structural domain (NICD) upon binding to ligands. NICD then translocates to the nucleus, where it binds to CBF1 (also known as *RBP-J* or CSL) and co-activators of Notch signaling (Maml), leading to the induction of target genes. These target genes include *Hes* family genes, particularly *Hes1* and *Hes5*, and genes belonging to the Hey family [60]. Repressive bHLH proteins, encoded by these genes, inhibit the activity of pro-neural bHLH proteins such as *Ascl1* in the ventral forebrain and *Neurog1/2* in the neocortex [60], inhibiting cells containing Notch signaling from becoming neurons.

Reduction of Notch signaling drives reactive astrocytes from the striatum to initiate neurogenesis following ischemic stroke, and further blocking *Rbpj* allows more astrocytes to initiate expression of the pro-neural transcription factor Ascl1 and progressively expressing neuronal maturation markers [15,61]. Single-cell sequencing has shown that after Notch signaling blockade, astrocytes in the striatum and cortex initiate the same neurogenic program in the transcriptome as they do in the subventricular zone [62]. *Dlx2* may trigger *Ascl1*+ cell production in striatal astrocytes by inhibiting the Notch pathway, which normally inhibits pre-neural programs. This inhibition may lead to the up-regulation of *Ascl1* expression in a cell-autonomous manner, inducing astrocyte-to-neuronal conversion [31]. Furthermore, blocking the Notch signaling pathway by the small molecule DAPT (a γ-secretase inhibitor) alone or in combination with other small molecules also reprograms astrocytes into functional neurons [41,42,63].

### BMP/Smad signaling pathway

BMP is a member of the TGF-β superfamily that is widely present in several biological systems. During early development of the nervous system, the BMP signaling pathway is involved in many important developmental events, such as formation of the neural tube, control of the number and distribution of neuronal cells, differentiation and apoptosis of embryonic neurons, and regulation of the ratio of neurons to glial cells in the nervous system [64]. During the late embryonic and postnatal periods, BMP/Smad signaling strongly induces astrocyte differentiation [65]. Smad proteins are major intracellular signaling sensors for BMP receptors and initiate the canonical BMP signaling pathway by activating Smad 1, 5, and 8 when BMP ligands bind to the receptor [66]. BMP signaling promotes *Ascl1* degradation, thereby promoting gliogenesis and inhibiting neurogenesis [67]. The combined suppression of BMP/Smad signaling by Noggin and LDN193189 has been demonstrated to initiate a rapid and comprehensive neural transition in human embryonic stem cells [68]. The addition of Noggin during *Sox2* induction in striatal astrocytes to neuroblasts further promotes neuronal maturation and enables integration into local neural networks [37]. In addition, LDN193189 in combination with other small molecules, successfully reprograms human astrocytes into neurons [41,42] and transforms mouse spinal cord astrocytes into motor neurons [69].

### Wnt/GSK-3 signaling pathway

Wnt proteins are secreted glycoproteins identified as key regulators of regional identity in the early developing forebrain [70]. The Wnt/GSK-3 signaling pathway plays a role in regulating both postnatal and adult neurogenesis [71], and it is involved in promoting neuronal differentiation and regulating cellular self-renewal pluripotency [33]. The activation of the Wnt/β-catenin pathway inhibits astrocyte differentiation by upregulating *Ngn2* expression [72]. After cerebral ischemia, Wnt2 released from apoptotic neurons activates Wnt/β-linked protein signaling in reactive astrocytes triggering astrocyte dedifferentiation, thereby promoting cortical neurogenesis [73]. Chen and Yin et al. inhibited GSK-3 indirectly to activate the Wnt pathway through treatment withCHIR99021 and kenpaullone and other small molecule compounds, thus activating the expression of *Ngn2* and promoting astrocyte-to-neuron transformation [39,42]. However, the GSK3 signaling pathway inhibitor CHIR99021 alone induced a 120-fold increase in *Ngn2* expression [42] but did not induce neurogenesis, suggesting crosstalk between various signaling pathways, which needs further elucidation.

### TGF-β signaling pathway

TGF-β plays a key role in regulating neuronal and glial cell survival, differentiation, proliferation, and activation [74]. Activated TGF-β receptors have been reported to stimulate phosphorylation of receptor-regulated Smad2 and Smad3 proteins after CNS injury, and phosphorylation of these Smad proteins leads to degradation of *Ascl1*, thereby promoting proliferation of reactive glial cells and glial scar formation [67]. RepSox, an inhibitor of the TGF-β signaling pathway, when combined with other small molecule chemicals such as VPA and CHIR99021, induces mouse astrocytes to withdraw from the cell cycle, activating the expression of *Ngn2* and *NeuroD1*, and directly converting astrocytes to neurons *in vitro*. Even the use of another small molecule targeting the same signaling pathway, such as trinostat (an anti-allergic drug) instead of RepSox, was successful in reprogramming mouse astrocytes into neuronal cells [39]. In addition, SB431542T, another TGF-β inhibitor, promoted astrocyte-to-neuronal conversion when mixed with other small-molecule compounds [42].

Along with the pathways previously mentioned, additional signaling pathways, including the JAK-STAT signaling pathway, transcription factor signaling, and transcriptional activator 3 (STAT3), are involved in the upregulation of astrocyte structural proteins following injury. This causes astrocyte skeletal remodeling and induces cellular hypertrophy, resulting in the formation of reactive astrocytes [12]. In addition, the p38 MAPK, mTOR, cAMP/PKA, cAMP/PKA, JNK [75], and p53 pathways [76] have been reported to be associated with astrocyte reprogramming into neurons. Therefore, selective inhibition of signaling pathways involved in gliogenesis and activation of signaling pathways involved in neurogenesis are essential for neuronal regeneration. Cross-talk exists between these signaling pathways, and simultaneous inhibition with the addition of nine small-molecule signaling inhibitors at the same time led to a failed reprogramming strategy, suggesting that some signaling pathways cannot be simultaneously inhibited during reprogramming [41].

In addition, inhibition of different signaling pathways using combinations of different small-molecule compounds may induce different efficiencies and neuronal subtypes in astrocytes in different populations of the brain; further studies are needed to explore the mechanisms of these signaling pathways.

## Reprogramming in neurological disorders

According to previous research, reprogramming astrocytes *in situ* surrounding lesions to become distinct neuronal subtypes may offer a therapeutic approach for neurological conditions. We summarize the current research on the induction of the different neuron subtypes in different models of neurological disorders (Figure 1). For instance, induction of dopaminergic neuron production can effectively restore symptoms in patients with PD [51,77], induction of GABAergic neuron production is effective in improving symptoms in patients with HD [34] and epilepsy [35], and induction of glutamatergic neurons partially relieves symptoms in patients with Alzheimer’s disease [32]. In addition, inducing motor neuron production can promote recovery from SCI as well as ALS [69].

### Strokes

The protection and regeneration of neurons after a stroke are crucial for promoting neurological recovery. Reprogramming reactive astrocytes in damaged areas of the brain into neurons, thus re-establishing functional connections between neurons, is a popular cell replacement therapy.

In 2014, Magnusson et al. found that mouse astrocytes accumulated *Ascl1* expression after middle cerebral artery occlusion (MCAO) and progressively expressed the immature neuronal marker DCX and the mature neuronal marker NEUN [15]. Furthermore, Duan et al. demonstrated that MCAO-induced reactive astrocytes might directly transdifferentiate to become neurons with the same ability to produce and release neurotransmitters as neurons in healthy brain tissue [78]. These results imply that a vast number of reactive activated astrocytes are a source of neural regeneration following stroke. In 2020, Chen et al. effectively restored motor and cognitive function in mice after a stroke by reprogramming reactive astrocytes in the infarct site into functioning neurons by overexpressing the *NeuroD1* gene using an adeno-associated virus (AAV) [23]. In addition, using a non-human primate ischemic stroke model, Ge et al. overexpressed NeuroD1 in astrocytes via AAV and successfully converted reactive astrocytes into functional neurons in areas of brain injury [24]. These results indicate that gene therapy may offer new treatment options for patients with stroke. Although considerable progress has been made in this field, some crucial problems remain. For example, a high incidence of stroke occurs in middle and old age. However, employing a retroviral delivery method that encodes the transcription factor Ngn2 alone or in combination with the anti-apoptotic protein Bcl-2 to target proliferating astrocytes in the neocortex of young or old mice resulted in very poor conversion efficiency to neurons. This could be as a result of the therapeutic vector encoding the altered gene being phagocytized after being administered intracortically [25]. Overall, more studies are needed to explore different gene combination approaches, safer and more effective delivery systems, and different treatment time windows.

### Parkinson’s disease

PD is a common neurodegenerative disease that is characterized by chronic degeneration and death of nigrostriatal dopaminergic neurons as its main pathological feature. This pathology causes brain motor neurons to generate less dopamine, which leads to motor dysfunction including trembling hands, stiffness, slow movement, and balance issues. Current treatments include medications, surgery, and physical therapy, which can control the condition and relieve symptoms but cannot cure the disease. Therefore, finding new treatments is presently an important research direction in the field of PD therapeutics [79]. PD is treated by converting astrocytes in the striatum into dopaminergic neurons to replace lost neurons. This provides a new approach for the treatment of neurodegenerative diseases. Astrocytes in the striatum were reprogrammed into dopaminergic neurons using lentiviral vectors overexpressing *NeuroD1*, *Ascl1*, Lmx1a and microRNA 218. In a mouse model of PD, the altered neurons not only produced action potentials but also significantly enhanced motor performance [27]. In a recent study, scientists used an inventive reprogramming method to successfully transform astrocytes into neurons by introducing the transcription factor combination of *Ascl1, Lmx1a, Nr4a2* (ALN), *Ascl1, Lmx1a, NeuroD1*, and miRNA218 (ALNe-218) using an endogenous peptide-split dCas9 activator system. The reprogrammed neurons exhibited maturity and typical neuronal properties, including generation of action potentials, whereas astrocytes did not. In a mouse model of PD, reprogrammed neurons alleviated the motor deficits caused by toxin exposure []. Additionally, by inhibiting the RNA-binding protein PTB, researchers have efficiently reprogrammed cortical astrocytes into mature neurons and converted them into dopaminergic neurons in mice. Researchers have used AAV-Cre technology to effectively restore striatal-mesenchymal pathway activity and significantly improve motor impairment in a PD animal model [51]. Similarly, researchers have found that injection of AAV-GFAP-CasRx-Ptbp1 into the striatum of a PD mouse model using the newly developed, highly specific, and efficient RNA-targeting CRISPR system CasRx resulted in a 10% reduction in the expression of the RNA-targeted PTBP1 gene. This effectively inhibits PTPB1 activity and leads to the conversion of astrocytes to dopaminergic neurons, thereby ameliorating Parkinson-like dyskinesia [52].

However, several concerns remain unresolved regarding direct *in situ* transformation of astrocytes into functional dopaminergic neurons. One concern is that PTB, which is inhibited to enable its conversion, is expressed in various cell types within the midbrain, including endothelial cells, pericytes, ventricular canal cells, and microglia. Therefore, it is unclear whether PTB inhibition affects dopaminergic neurons or other neuronal types in animal models of PD [80]. Furthermore, although more than half of the striatally projecting fibers are derived from induced dopaminergic neurons, most transformed dopaminergic neurons tend to innervate the septum rather than the striatum. This implies that further optimization of the transformation protocol may be required to improve the specificity of neuronal targeting [51,80].

Although regenerative medicine based on *in vivo* gene therapy strategies for neuronal replacement has great promise in the treatment of neurological diseases such as PD, caution is warranted, especially for human midbrain and striatal astrocytes. In addition to establishing that the altered cell types and their targeting are accurate and stable, more studies are required to evaluate whether such transformation is viable in humans. Further experimental and clinical studies are required to confirm the reliability and safety of this approach.

### Huntington’s disease

HD is a genetic disorder that causes progressive neurodegeneration in the brain, resulting in severe physical, cognitive, and mental impairments. The *Htt* gene mutations that cause this disease promote the buildup of aberrant proteins in the brain, including notable accumulation in the striatum. Currently, there is no cure for HD, and the available treatments can only alleviate the symptoms. However, ongoing research is aimed at developing new therapies designed to slow or stop disease progression, including gene therapy, stem cell therapy, and various pharmacological approaches [81]. The regeneration of functional neurons in aged mammalian brains by the direct reprogramming of glial cells into neurons is a novel cell replacement therapy for HD. In 2020, Wu et al. described a novel cell replacement therapy for HD that involved the direct reprogramming of glial cells in the aged mammalian brain into neurons. Through AAV-mediated ectopic expression of the transcription factors *NeuroD1* and *Dlx2*, researchers have successfully reprogrammed striatal astrocytes into functional GABAergic neurons. These newly transformed neurons not only showed action potentials and synaptic events but also significantly improved motor function and prolonged lifespan in HD model mice [34]. Although reprogramming glial cells into neurons is a promising cellular therapy for HD, it fails to address the underlying genetic mutations causing this disease. Transformed neurons may eventually suffer the same degeneration because of accumulation of the mutant Htt protein. One proposed solution is to combine cell transformation and CRISPR gene editing technology to correct mutations. By treating HD at the origin of the disease, transformed neurons may have a better chance of survival [34].

### Alzheimer’s disease

AD is a common neurodegenerative disorder that is characterized by the accumulation of beta amyloid, leading to neuronal and synaptic damage primarily located in cortical and hippocampal regions. The cognitive and behavioral issues brought on by this illness progress over time, resulting in language impairment, memory loss, cognitive decline, and significant decline in one's quality of life. Unfortunately, there is no cure for AD, although medication and behavioral interventions can help manage its symptoms [82]. Guo et al. effectively converted reactive glial cells into functional glutamatergic neurons in the cortex of AD model mice using a retrovirus expressing the transcription factor *NeuroD1*. Surprisingly, more inducible neurons were successfully generated in the aging AD model. These inducible neurons successfully integrated into primitive neural circuits, generating spontaneous and evocative synaptic responses, indicating successful establishment of synaptic connections with other neurons. In addition, these inducible neurons have morphological and electrophysiological characteristics similar to those of normal glutamatergic neurons [32]. The generation of these inducible neurons offer new hope for the treatment of AD. Unfortunately, this article does not clarify whether reprogramming glial cells into neurons *in vivo* can ultimately rescue behavioral deficits such as mental impairment and memory loss in mouse models of AD and fails to elucidate the effect of different age groups on the reprogramming efficiency of astrocytes. Additionally, before undertaking preclinical and clinical investigations, it is important to carefully consider the long-term and possible negative effects of these generated cells on relevant neurological systems.

### Epilepsy

Epilepsy is a chronic neurological disorder that affects the brain and is characterized by recurrent seizures. Temporal lobe epilepsy (TLE) is a common form of epilepsy that is characterized by decreased GABAergic inhibition in the brain and increased excitability of temporal lobe neurons, which leads to spontaneous recurring seizures [83]. Antiepileptic drugs are currently the primary treatment for epilepsy, suppressing seizures by affecting neuronal activity. However, existing antiepileptic drugs have limited efficacy in some patients, with drug resistance developing in approximately 30–40% of cases [84]. Therefore, there is a need for more effective and individualized epilepsy treatment options in addition to antiepileptic drugs. Chen et al. reported a promising approach to treating TLE through transdifferentiation of astrocytes into functional neurons. Researchers delivered *NeuroD1* into reactive astrocytes in the hippocampus of rats with TLE using an AAV vector. This induces the conversion of astrocytes into GABAergic neurons, and the newly generated GABAergic neurons were found to integrate into hippocampal circuits and suppress spontaneous recurrent seizures. Furthermore, these neurons improved cognitive and emotional function in a rat model of TLE [35]. In 2022, Lentini et al. developed a model of medial temporal lobe epilepsy with hippocampal sclerosis (MTLE-HS) by injecting kainite (KA) into the hippocampal region of adult mice. Reprogramming reactive glia into interneurons reduces chronic seizure activity in a mouse model of mesial lobe epilepsy. Targeting reactive glial cells in the epileptic hippocampus by retroviruses carrying the *Ascl1* and *Dlx2* genes converts them into neurons, and these newborn neurons integrate into neural networks and establish GABAergic synapses on dentate granule cells and also reduce the frequency and severity of spontaneous recurrent hippocampal seizures in MTLE-HS mice [85]. Thus, cellular reprogramming of glial cells to generate inhibitory GABAergic neurons is a promising cell-replacement therapy for the treatment of epilepsy. However, there is still a long way to go before these findings are translated into a clinical setting. Hippocampal reactive astrocytes proliferate along with epilepsy, but it is unclear at what stage of the disease there will be enough dividing glial cells to be therapeutically beneficial. If all local glial cells are severely damaged, it may also be difficult to regenerate functional new neurons in the damaged areas. Finally, astrocytes can proliferate, differentiate, and produce various signaling molecules that regulate the interaction between neurons and glial cells in different states. Therefore, when selecting viral vectors, the optimal timing of the intervention should be determined based on the proliferation status of the target cells and the possibility of creating a ratio imbalance should be closely monitored to ensure the effectiveness and safety of the intervention.

### Spinal cord injury

SCI results in sensory, motor, and autonomic nervous system dysfunction owing to axonal damage and neuronal death brought on by several physiochemical causes. Presently, SCI is still an incurable condition; therefore, researchers have been exploring ways to help patients regain function. Some of these methods include using stem cells and medications that promote neural regeneration [86]. Currently, one of the most promising approaches is to exploit the plasticity of astrocytes and convert them into neurons *in vivo* to replace neurons lost after SCI [87]. In addition, reactive glial cells proliferate after damage and cause glial scarring, which can prevent nerve regeneration in the injured areas. Converting these glial cells into neurons can directly reduce the number of reactive glial cells, and thus limit the amount of glial scarring [88]. In a recent study, researchers found that *Sox2* promotes the conversion of astrocytes to neurons in the spinal cord. Its use in combination with VPA can further convert these neurons into GABA-like neurons. In addition, these newly formed neurons can form synaptic connections with existing neurons [38]. In recent years, a series of successful *in vivo* conversions of astrocytes into neurons have been achieved through AAV-mediated expression or activation of exogenous neurotranscription factors, as well as CRISPR-mediated expression or activation of endogenous genes. For example, through AAV-mediated expression of *NeuroD1* [36] or *Ngn2* [89], researchers have successfully transformed astrocytes into different types of neurons capable of forming synaptic connections and generating multiple action potentials. Similar transformations have been achieved through the CRISPR-mediated activation of *Ngn2* and Isl1, and these newly formed neurons are also capable of projecting to the sciatic nerve [22]. Furthermore, by blocking NOTCH1 signaling with a single small molecule, Tan et al. found that reactive astrocytes could be converted into neurons after SCI but not with high efficiency [63].

Although astrocyte reprogramming offers new directions and hope for neurons to treat SCI, it is currently unclear how long these newly formed neurons survive *in vivo*, whether they generate the correct neuronal subtypes, and whether they can connect efficiently with the surrounding neural circuits to restore function. In addition, the methods of these transformations still need further refinement to improve their efficiency and specificity and to reduce potential safety risks.

### Amyotrophic lateral sclerosis

Amyotrophic lateral sclerosis (ALS) with progressive loss of muscle function and control is a rare but dangerous neurodegenerative disease that primarily affects upper and lower motor neurons in the motor cortex, brainstem, and spinal cord [90]. The pathogenesis of the disease is not fully understood. Currently, there is no clinical cure for ALS, and only medications and physical therapy are available to relieve the symptoms. In a recent study, the use of identified small molecule cocktails (pegylone, forskolin, vincristine and retinoic acid), Zhao et al. effectively transformed human astrocytes into motor neuron-like cells *in vitro*, and these induced motor neuron-like cells expressed motor neuron markers and exhibited the electrophysiological properties of neurons. Interestingly, investigators extended this technique to a mouse ALS model carrying the SOD1 mutation and achieved the same transformation [69]. The success of this technique may provide new ideas and approaches for ALS treatment. However, some uncertainties remain. One important question is whether these induced cells can integrate into the correct neural circuits in the brain. For example, will they seek and connect to their natural target cells or will they connect to wrong neurons, leading to unexpected effects or side effects? In addition, another key question is whether these induced cells are effective in improving behavioral deficits and promoting the recovery of brain function. Although some studies have demonstrated that these induced cells can improve symptoms in mouse models, whether this effect can be maintained and extended in higher-level animal models and eventually translated into a treatment for human patients requires further exploration.

## Challenges and prospects

Conversion of glial cells into neurons has become a popular research topic in regenerative medicine. Because of their wide distribution, abundance, easy access, and efficient differentiation, glial cell transformation into neurons is considered a promising therapy. Although this technology has been successful in mice and human cells, challenges remain to be addressed before clinical application.

### Securing newborn neurons from astrocytes

A key problem in the conversion of astrocytes into neurons is the ability to distinguish between reprogrammed and pre-existing neurons. One possible solution is to use transgenic strategies to label specific cell populations and track their origins during transformation to distinguish between pre-existing neurons and iNs. The current use of spectral tracking mouse lines, such as the Aldh1l1-CreERT2; R26R-YFP and mGfap-Cre; R26R-YFP mouse lines using tamoxifen-induced, specifically labeled astrocytes, allows for a thorough examination of the cell-type specificity of genetic reporter genes. Second, glial-to-neuronal conversion is a cellular process that involves multiple intermediate/transient states, and the conversion of glial cells to neurons can be observed using delayed imaging to clarify the reprogramming process. Additionally, some proliferation markers like EdU and BrdU, insert thymidine nucleoside analogs into the DNA of dividing cells during the S phase of the cell cycle. These cells can be identified using antibody- or non-antibody-based methods. Because endogenous neurons are postmitotic, they cannot be tracked using this method [91]. Finally, with the development of single-cell sequencing, scRNA-seq has been used in combination with genealogical tracing to detect cells of origin while monitoring the transcriptional process of fate transition and the identity of emerging neurons [55].

### Neuronal subtypes and regional specificity

It has been reported that there is regional heterogeneity in astrocytes, such that in the cerebral cortex, *Ascl1* mainly induces GABAergic neurons, whereas *Ngn2* mainly induces glutamatergic neurons [46]. Furthermore, *Ngn2*-induced neurons in the neocortex exhibit glutamatergic properties, whereas neurons in the striatum are mainly striatal projection neurons [21]. Furthermore, the combination of *Ngn2* and Nurr1 results in efficient astrocyte reprogramming in the gray matter, and no astrocyte transdifferentiation is observed in the white matter [92]. Additionally, the effectiveness of reprogramming varies depending on the environment. For example, in the AD model, reactive astrocytes are reprogrammed more effectively in older than in young AD mice [32], indicating that reprogramming is influenced by both intrinsic cellular mechanisms and particular environmental cues. Thus, there is an evident need for a better understanding of the cellular and molecular mechanisms by which the local environment directs glial-to-neuronal transdifferentiation. Furthermore, for *in vivo* reprogramming, it is of utmost importance to ensure that astrocyte-transformed neurons are neurons that are lacking in the damaged region and that these nascent neurons are properly connected to pre-existing circuits.

### Improved reprogramming efficiency

For CNS repair, it is important to generate a sufficient number of functional neurons for repair to take place. According to reports, astrocyte reprogramming is ineffective, and a significant portion of newly produced neurons die soon after neuronal induction [20]. Therefore, improving reprogramming and ensuring the maturation as well as prolonged survival of iNs are urgent questions that need to be answered. Conventional astrocytes are energized by glycolysis, whereas neurons are activated by oxidative phosphorylation and undergo oxidative metabolic conversion during the reprogramming of astrocytes into neurons. Neonatally iNs undergo iron death due to increased lipid peroxidation, which is caused by a late switch or deletion in the mitochondrial proteome [93]. According to some studies, the antiapoptotic gene Bcl-2 [20] and antioxidants like vitamin C [94], or the small molecule compounds VPA, osteotrienols, or -tocotrienols [38] have increased neuronal conversion efficiency and lifespan. Furthermore, the transformation of astrocytes into the iNs lineage is enhanced by the co-expression of *Ascl1* with the neuron-enriched antioxidant genes *Sod1* and *Prdx1* [93]. Accordingly, further research is needed to overcome the barriers to reprogramming, to promote safer transitions between cell fates, and to improve efficiency.

### Delivery systems

Most of the *in vivo* delivery of transcription factors and miRNAs depends on viral vectors, and the genomic integration ability of retroviruses and lentiviruses may lead to tumorigenesis, raising inevitable questions about their safety. Low immunogenicity-based AAV vectors are now the preferred method for gene therapy; however, their low transfection efficiency requires increased viral titers, and leakage may occur with increasing viral titers [36]. Therefore, further studies are needed to explore the appropriate AAV titers. In contrast, reprogramming induced by small-molecule compounds has received much attention because they do not integrate into genes and are easy to synthesize. However, more studies are needed to explore the optimal combinations and simplify induction procedures because of the wide variety and complexity of existing methods. In addition, how to reduce the side effects associated with systemic drug administration and how to make these drugs cross the blood–brain barrier efficiently and reach the correct sites must be determined. Recent studies have shown that novel intracellular delivery techniques combining small-molecule drugs such as LDN193189, SB431542, and DAPT with VPA can effectively convert astrocytes into mature GABAergic and glutamatergic interneurons; therefore, novel intracellular delivery techniques are expected to be a therapeutic tool for regenerative medicine [95]. Consequently, safe and reliable *in vivo* delivery systems need to be developed in the future.

## Conclusion

Overcoming the inherent challenges to neural reprogramming can unlock new possibilities for treating neurological injuries and offer hope to patients. In recent years, with the continued development and application of single-cell technologies, individual cell populations have been studied in greater detail. This technological advancement has allowed us to better understand and explore the process of cellular transformation and gain insights into the molecular mechanisms and pathways through which glial cells are reprogrammed into neurons. In addition, the development of *in vivo* tracking technologies, continued development of novel delivery systems, and application of small molecules will allow for greater clarity regarding the origin of reprogrammed cells and advance the field with safer, noninvasive, and more efficient reprogramming protocols.

## Abbreviations

AAV: Adeno-associated virus
AD: Alzheimer’s disease
ALS: Amyotrophic Lateral Sclerosis
bHLH: Basic helix-loop-helix
CNS: Central nervous system
CRISPRa: CRISPR-Cas transcriptional activation
DCX: Doublecortin
HD: Huntington’s disease
iNs: induced neurons
iPSC: Induced pluripotent stem cell
MCAO: Middle cerebral artery occlusion
OPC: Oligodendrocyte precursor cell
PD: Parkinson’s disease
SCI: Spinal Cord Injury
TLE: Temporal lobe epilepsy
VPA: Valproic acid
