# Correction: A promise for neuronal repair: reprogramming astrocytes into neurons in vivo

**DOI:** 10.1042/BSR-2023-1717_COR

**Published:** 2024-07-03

**Keywords:** astrocytes, neurodegeneration, neurogeneration, transcription factors

The authors of the original article “A Promise for Neuronal Repair: Reprogramming Astrocytes into Neurons in Vivo” (doi: 10.1042/BSR20231717) would like to correct Figure 1.

**Figure 1:**
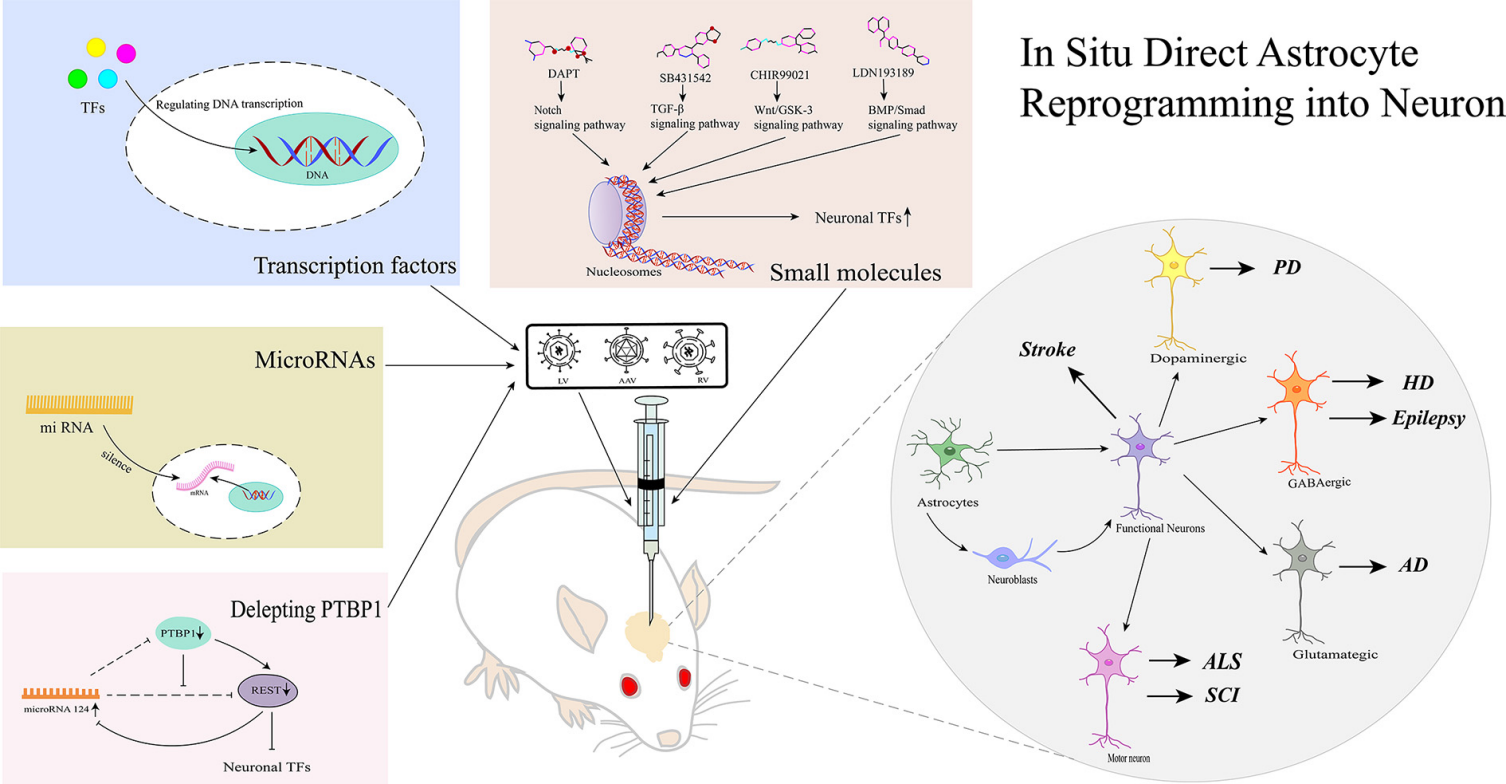
Methods for reprogramming astrocytes into neurons *in* *vivo*

After publication, the authors identified that “PTBP1” in the Figure was incorrectly written as “PTB1”. The corrected figure appears below.

Additionally, within the main text under the “Depleting PTBP1” section, “… Qian et al. delivered an RNA-silencing hairpin (shRNA) using AAV2 in vivo to reduce PTB1 expression in astrocytes of GFAP-Cre mice” should instead read “… Qian et al. delivered an RNA-silencing hairpin (shRNA) using AAV2 in vivo to reduce PTBP1 expression in astrocytes of GFAP-Cre mice”.

The authors declare that this correction does not change the results or conclusions of their paper.

